# 0099. Nitrite reductase activity during sepsis

**DOI:** 10.1186/2197-425X-2-S1-P10

**Published:** 2014-09-26

**Authors:** V Simon, A Dyson, M Minnion, M Feelisch, M Singer

**Affiliations:** University College London Hospital NHS Foundation Trust, Bloomsbury Institute of Intensive Care Medicine, London, UK; University Hospital Aachen, Intensive Care Medicine, Aachen, Germany; University of Southampton, Faculty of Medicine, Southampton, UK

## Introduction

Nitric oxide (NO) excess is considered to be the main cause of hypotension in sepsis. Research has mainly focussed upon NO production by isoforms of NO synthase (NOS). However, alternative pathways may make an important contribution, including nitrite (NO_2_^-^) reduction by the reductase activity possessed by numerous heme- and pterin-based enzymes. The temporal contribution of these pathways to NO production in sepsis is currently unclear.

## Objectives

To investigate changes in nitrite reductase activity during sepsis.

## Methods

Male Wistar rats (approx 300g wt) with tunnelled right jugular venous and left common carotid arterial lines *in situ* received i.p. injection of either faecal slurry (septic) or n-saline (sham). Fluid (1:1 mixture of 5% glucose/Hartmann`s; 10 ml/kg/h) was started 2h later. At either 6h or 24h, rats were anaesthetized and tracheotomized. After stabilization, measurements were made of haemodynamics (blood pressure, echocardiography) and methaemoglobinaemia (oxidation of Hb to the Fe^3+^ form induced by NO or nitrite) before and after a 25 ml/kg bolus fluid challenge (BL, baseline) to ensure adequate LV filling. Animals then received (A) a single dose of sodium nitrite (NaNO_2_, 15mg/kg i.v.) to stimulate nitrite reductase activity, (B) a combination of NaNO_2_ and the NO-scavenger cPTIO (3.4mg/kg i.v.) or (C) a combination of NaNO_2_ and the non-specific NOS-inhibitor SEITU (1mg/kg i.v.). Recordings were made over the next 2-4h prior to sacrifice.

## Results

**Figure 1:**
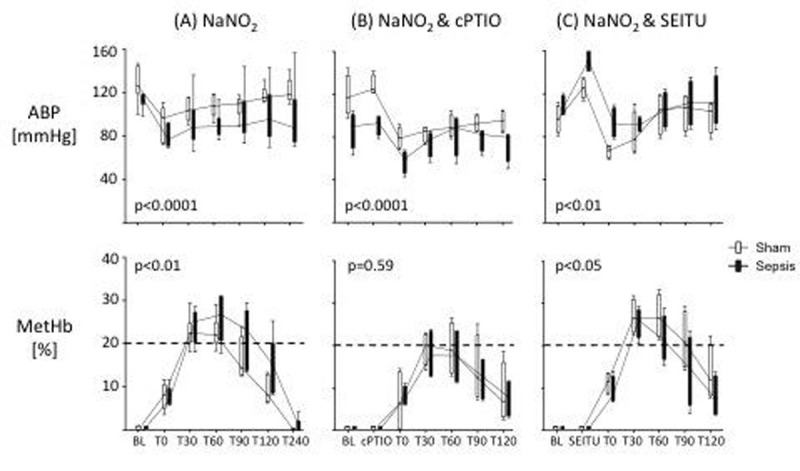
Effects of NaNO_2_, cPTIO and SEITU on arterial blood pressure (ABP) and methaemoglobin levels (MetHb) in sham and septic animals at 24h. BL, baseline; T0, after drug administration (2-way ANOVA + Bonferroni test, *p< 0.05).

At 6h, there was no significant difference between groups (data not shown). However, at 24h, septic animals showed a greater fall in BP following NaNO_2_ with a longer time to recovery, signifying increased nitrite reductase activity (Fig 1A). After scavenging of free NO (1B) and NOS inhibition (1C), both groups showed an initial rise in BP, followed by a significant fall with NaNO_2_. However, on removal of NO there was no prolonged BP recovery time in the septic animals. Baseline methaemoglobinaemia was low in both control and septic animals at 24h and increased markedly with NaNO_2_, again with a longer recovery time in septic animals. This rise was blunted by pre-treatment with the NO scavenger, c-PTIO, but not by SEITU though SEITU did prevent the delayed normalization in metHb levels .

## Conclusions

In this rat model, increased nitrite reductase activity was apparent during established (24h) sepsis, but not at an early (6h) timepoint. The clinical relevance of this finding needs to be elucidated.

